# Connexin 43 (Cx43) regulates high-glucose-induced retinal endothelial cell angiogenesis and retinal neovascularization

**DOI:** 10.3389/fendo.2022.909207

**Published:** 2022-09-02

**Authors:** Wen Shi, Zhishang Meng, Jing Luo

**Affiliations:** ^1^ Department of Ophthalmology, The Second Xiangya Hospital, Central South University, Changsha, China; ^2^ Department of Ophthalmology, Hunan Clinical Research Center of Ophthalmic Disease, Changsha, Hunan, China

**Keywords:** diabetic retinopathy, retinal neovascularization, human retinal endothelial cells, angiogenesis, connexin 43 (Cx43)

## Abstract

Diabetic retinopathy (DR) is an important microvascular complication of type 1 and type 2 diabetes mellitus (DM) and a major cause of blindness. Retinal neovascularization plays a critical role in the proliferative DR. In this study, high glucose-induced connexin 43 (Cx43) expression in human retinal endothelial cells (hRECs) in a dose-dependent manner. Compared with hRECs under normal culture conditions, high-glucose (HG)-stimulated hRECs showed promoted tubule formation, increased ROS release, and elevated levels of tumor necrosis factor-alpha (TNF-α), interleukin-1β (IL-1β), vascular endothelial growth factor A (VEGFA), and intercellular adhesion molecule 1 (ICAM-1) in the culture medium. HG-induced alterations were further magnified after *Cx43* overexpression, whereas partially eliminated after *Cx43* knockdown. Finally, in the DR mouse model, impaired retinal structure, increased CD31 expression, and elevated mRNA levels of *TNF-α*, *IL-1β*, *VEGFA*, and *ICAM-1* were observed; *in-vivo Cx43* knockdown partially reversed these phenomena. Conclusively, *Cx43* knockdown could inhibit hREC angiogenesis, therefore improving DR in the mouse model.

## Introduction

Diabetic retinopathy (DR) is an important microvascular complication of type 1 and type 2 diabetes mellitus (DM), which makes great contributions to blindness (1, 2). Due to the increasing prevalence of DM, DR is increasingly widespread and endangers the vision of patients (3–5). For diabetic tractional retinal detachment in proliferative DR, an advanced form of DR, pars plana vitrectomy is typically performed to repair vision or avoid additional visual loss (6).

The pathology of DR is complex, involving retinal inflammation, increased retinal vascular permeability, and blood-retinal barrier breakdown (7). These malignant processes result in the uncontrolled proliferation and migration of retinal microvascular endothelial cells and the formation of capillary, leading to retinal neovascularization (8). It has been pointed out that retinal neovascularization is one of the most common causes of retinal detachment, vitreous hemorrhage, and sight loss in the proliferative DR (9). Therefore, understanding the underlying mechanisms of retinal neovascularization may provide an effective therapeutic approach for DR. Vascular endothelial growth factor (VEGF) has been proposed as a potent mediator of DR angiogenesis, and VEGF-targeting drugs (such as ranibizumab and aflibercept) have shown promising results (10). Nevertheless, other pro-inflammatory and pro-fibrotic mediators [such as tumor necrosis factor-α (TNF-α), monocyte chemotactic protein 1 (MCP-1), and intercellular adhesion molecule-1 (ICAM-1)] are evidenced to be involved in anti-VEGF therapeutic failure (11). Therefore, more mechanisms of retinal neovascularization through retinal endothelial cells (RECs) in DR must be further validated.

Intercellular communication has been evidenced to contribute greatly to vascular function and homeostasis. Connexins (Cxs) exert crucial effects on this interaction. Cxs, cataloged as transmembrane proteins, form hemichannels in the plasma membrane and intercellular channels, termed gap junctions, connecting the cytoplasm of two adjacent cells (12, 13). As has been reported previously, on vascular walls, 4 Cxs (Cx37, Cx40, Cx43, and Cx45) are found within endothelial cells and smooth muscle cells (12, 13). Endothelial cell proliferation and migration are needed for angiogenesis, finally forming stabilized vessels by recruiting mural cells. Accumulating studies have shown that endothelial Cxs are tightly implicated in modulating these processes (14–17). Knocking out endothelial Cxs reduces angiogenesis (18). On the contrary, upregulated endothelial Cxs (such as Cx37, Cx43, and Cx40) are linked to angiogenesis induction (19, 20). Multiple studies have evidenced that Cx43 is essential for the positive regulation of endothelial cell and endothelial progenitor cell (EPC) migration (21–24). Consistently, Cx43siRNA treatment notably inhibited human late EPC abilities of migration and angiogenesis; within a mouse ischemic hind limb model, the therapeutic angiogenesis capacity of these EPCs is lost (25). Moreover, Cx43 expression is consistent with the increased levels of representative angiogenic factors in rat mesenchymal stem cells with Cx43 overexpression (26). Taken together, the above findings indicated a strong potential of endothelial Cx43 in promoting angiogenesis.

In this study, human RECs (hRECs) were first stimulated with high-glucose (HG) to simulate the DR microenvironment. Cx43 expression was validated in hRECs under normal or HG conditions. hREC tubule formation, reactive oxygen species (ROS) release, and the levels of inflammatory and angiogenesis markers (TNF-α, IL-1β, VEGFA, and ICAM-1) were investigated in normal or HG-treated hRECs. Cx43 overexpression/knockdown was achieved in normal or HG-treated hRECs to explore the *in-vitro* effects of Cx43 overexpression/knockdown on angiogenesis. Finally, the DR mouse model was induced; Cx43 knockdown was achieved through lentivirus infection to evaluate the *in-vivo* effects of Cx43 knockdown on DR angiogenesis. During diabetic retinopathy, high glucose causes endothelial cells to undergo a bidirectional activation of the inflammatory and epigenetic machinery, which eventually results in chronic inflammation and vascular problems, causing globally high morbidity and mortality (27, 28). Therefore, our findings identify *Cx43*siRNA as a druggable target for DR *via* regulating endothelial angiogenesis.

## Materials and methods

### Cell lineage and cell culture

hRECs (Cat. #6530, ScienCell, Carlsbad, CA, USA) were cultured (37°C, 5% CO_2_) in Endothelial Cell Medium (ECM, Cat. #1001, ScienCell) containing 5% fetal bovine serum (FBS; Cat. #0025, ScienCell). hRECs were allocated into four groups: normal, low-HG (culture medium containing 20 mmol/L glucose), middle-HG (culture medium containing 60 mmol/L glucose), and high-HG (culture medium containing 120 mmol/L glucose).

### Immunoblotting

Cells were collected using 0.05% Trypsin-EDTA (Thermo Scientific, Waltham, MA, USA). After phosphate-buffered saline (PBS) rinsing, cells were lysed using radioimmunoprecipitation assay (RIPA) buffer (Beyotime, Jiangsu, China). Protein concentration was measured using a BCA Protein Assay kit (Beyotime). Equivalent quantities of protein (30 µg) were injected and then loaded onto 12% sodium dodecyl sulfate-polyacrylamide gel electrophoresis (SDS-PAGE) for separation, followed by the transferring of gels onto polyvinylidene difluoride (PVDF) membranes (Millipore, Billerica, MA). After that, the membranes were blocked in 5% non-fat milk for 1 h at room temperature and then incubated (4°C, overnight) with primary antibodies anti-Cx43 (#3512, Cell Signaling Technology, Danvers, MA, USA) and Tubulin (11224-1-AP, Proteintech, USA). Following 1-h incubation (room temperature) with horseradish-peroxidase (HRP)-conjugated secondary antibodies. Finally, the blots were visualized using Super Enhanced Chemiluminescence (ECL) Detection Reagent (Beyotime), and the signals were analyzed using ImageJ software. Chemiluminescence was determined using a Tanon 5200 Chemiluminescent Imaging System (Tanon, Shanghai, China).

### Tubule formation assay

Tube formation assay was performed on Matrigel (BD Biosciences, Franklin Lakes, NJ, USA) to assess angiogenesis. In brief, 10 mg/ml Matrigel (50 µl/well) in serum-free media was applied to 96-well plates, followed by a 30-min incubation at 37°C. HG-stimulated or transfected cells were seeded onto Matrigel-coated plates and then incubated (6 h, 37°C) in the cell culture incubator. Photographs obtained using a light microscope (Olympus, Japan). The tubule formation in each image was quantified using ImageJ software.

### Flow cytometry detecting ROS content

ROS production in hRECs was detected using the Reactive Oxygen Species Assay Kit (Beyotime). Specifically, live cells underwent 30-min incubation (37°C) in the serum-free culture medium containing 10 mM 2,7-dichlorofluorescein diacetate (DCFH-DA; Beyotime) or PBS (as blank) in the dark for detecting intracellular ROS level. Next, after trypsinization, the cells were rinsed again in PBS and resuspended (1×10^6^ cells/mL) in a serum-free medium for flow cytometry analysis (Novocyte, Agilent, USA). The fsc/ssc scatters were shown in **Figure S1**.

### Enzyme-linked immunosorbent assay

The protein contents of hRECs-released TNF-α, IL-1β, VEGFA, and ICAM-1 in the conditioned medium were determined using corresponding commercially available human ELISA kits (CUSABIO, China). The conditioned medium was harvested and subjected to 15-min centrifugation (350 × g, 4°C) for cellular debris removal. After that, the conditioned medium was recollected and maintained at -80°C for a subsequent ELISA assay.

### Cell transfection

The vector containing shRNA for the human *Cx43* gene (sh-Cx43#1/2/3; GenePharma, Shanghai, China) and/or human *Cx43* gene coding sequence (Cx43 OE, GenePharma) were obtained for cell transfection. Cells were transfected after growing to 60% density in culture plates. Next, hRECs were transfected with knockdown/overexpression vector (final concentration 2 μg/ml) using lipo3000 (5 μl/well, Invitrogen, USA). After 48 h, the cells were harvested for the subsequent experiments. The primers for vector sh-CX43#1/2/3 and CX43OE construction were listed in **Table 1**.

**Table 1:** The sequence of RT-PCR primer and shRNA.

RT-PCR primer (mouse)	Forward 5’-3’	Reverse 5’-3’
CD31	ACCGGGTGCTGTTCTATAAGG	TCACCTCGTACTCAATCGTGG
TNF-α	CCCTCACACTCAGATCATCTTCT	GCTACGACGTGGGCTACAG
IL-1β	CTGTGACTCATGGGATGATGATG	CGGAGCCTGTAGTGCAGTTG
VEGFA	CGATGAAGCCCTGGAGTGCGT	ACAAACAAATGCTTTCTCCGCTCTG
ICAM-1	GGCATTGTTCTCTAATGTCTCCG	TGTCGAGCTTTGGGATGGTAG
Tubulin	GCATTAACTACCAGCCTCCCAC	CGCCTTCCACAGAATCCACAC
Vector construction primer (human)	Forward 5’-3’	Reverse 5’-3’
Cx43 overexpression	CTACCGGACTCAGATCTCGAGATGGGTGACTGGAGCGCC	GTACCGTCGACTGCAGAATTCCTAGATCTCCAGGTCATCAGGCC
Sh-NC	GATCCGCCAAGCCCTTGTCTTCACAACTCGAGTTGTGAAGACAAGGGCTTGGCTTTTTG	AATTCAAAAAGCCAAGCCCTTGTCTTCACAACTCGAGTTGTGAAGACAAGGGCTTGGCG
Sh-Cx43 1	GATCCGTATTGAAGAGCATGGTAAGGCTCGAGCCTTACCATGCTCTTCAATACTTTTTG	AATTCAAAAAGGATTGAAGAACACGGCAAGGCTCGAGCCTTACCATGCTCTTCAATACG
Sh-Cx43 2	GATCCGGGTCCTGCAGATCATATTTGCTCGAGCAAATATGATCTGCAGGACCCTTTTTG	AATTCAAAAAGGGTCCTGCAGATCATATTTGCTCGAGCAAATATGATCTGCAGGACCCG
Sh-Cx43 3	GATCCGCCACTAGCCATTGTGGACCACTCGAGTGGTCCACAATGGCTAGTGGCTTTTTG	AATTCAAAAAGCCACTAGCCATTGTGGACCACTCGAGTGGTCCACAATGGCTAGTGGCG
Vector construction primer (mouse)	Forward 5’-3’	Reverse 5’-3’
lv-sh-Cx43	GATCCGGGTCCTTCAGATCATATTCGCTCGAGCGAATATGATCTGAAGGACCCTTTTTG	AATTCAAAAAGGGTCCTTCAGATCATATTCGCTCGAGCGAATATGATCTGAAGGACCCG
lv-Sh-NC	GATCCGCCAAGCCCTTGTCTTCACAACTCGAGTTGTGAAGACAAGGGCTTGGCTTTTTG	AATTCAAAAAGCCAAGCCCTTGTCTTCACAACTCGAGTTGTGAAGACAAGGGCTTGGCG

### Establishment of DR model of mice

A total of 24 C57BL/6 J mice (6 weeks, 23 ± 2.34 g, male) procured from Changsha SLAC laboratory animal company (Changsha, China) were kept under 12/12 light/dark cycles, with free access to a normal chow diet. All the animal experimental procedures were conducted in compliance with the principles of laboratory animal care (National Institutes of Health, Bethesda, MD, USA) and Use of Animals in Ophthalmic and Vision Research and under the approval of the Second Xiangya Hospital of Central South University (approval number:2018005). Mice were randomly allocated into 4 groups (n=6): normal, DR model, DR + Lv-sh-NC, and DR + Lv-sh-Cx43.

Mice were intraperitoneally injected with 50 mg/kg streptozotocin (STZ) every day for 5 consecutive days for DR model induction. Mice were examined for blood glucose once a week. After STZ injection for 1 to 4 weeks, hyperglycemia occurred; after STZ injection for 16 weeks, mice were sacrificed for tissue collection.

At weeks 4, 8 and 12, and 16 after STZ injection, mice in the DR + Lv-sh-NC and DR + Lv-sh-Cx43 groups were intravitreally injected with sh-NC or sh-Cx43 lentivirus (2×10^6^ TU lentivirus in 1 μL, GeneChem, Shanghai, China) in the right eye. Briefly, after mouse eyes were dilated with compound tropicamide, the needle was inserted 1 mm outside the corneal limbus using a microsyringe for intravitreal injection. Mice were anesthetized with isoflurane (3%) in oxygen and underwent intravitreal injection.

### Hematoxylin and eosin staining

Mice were sacrificed under anesthesia. The enucleated eyes were fixed overnight in 4% paraformaldehyde at ambient temperature, paraffin-embedded, and then sectioned (5 μm). Next, H&E staining was performed.

### Immunofluorescent staining

Tissue samples were fixed in 4% paraformaldehyde, dehydrated with an alcohol gradient, paraffin-embedded, and sectioned. After deparaffinization, the sections were incubated (4°C, overnight) with a primary anti-CD31 antibody (11265-1-AP, Proteintech), and washed 3 times in PBS followed by a 1-h incubation (room temperature) with Cy5-labeled secondary antibody (Beyotime). After PBS washing, the sections underwent 5-min incubation in DAPI (Sigma-Aldrich, USA). Finally, the coverslips were mounted with glycerol, and fluorescence microscopy (Olympus) was applied for analysis.

### Quantitative reverse transcription-polymerase chain reaction

Total RNA was isolated from target tissues or cultured cells using TRIzol reagent (Invitrogen) as per the manufacturer’s instructions. cDNAs were synthesized using the M-MLV Reverse Transcriptase Kit (ELK Biotechnology, Wuhan, China) for mRNA expression assessment. Real-time PCR was performed using a StepOne™ Real-Time PCR instrument (Thermo Scientific). Tubulin served as a loading control to measure the mRNA level, and the 2^−ΔΔCt^ method was used for calculation. The primer sequence was listed in **Table 1**.

### Statistical analysis

All data were expressed as mean ± standard deviation (SD). GraphPad Prism (GraphPad Software, Inc., La Jolla, CA, USA) was employed for data analysis. Two-group comparisons were analyzed using Student’s *t*-test; multiple comparisons were analyzed using one-way ANOVA with Tukey’s *post-hoc* test. *P*<0.05 was considered statistically significant.

## Results

### HG impairs human retinal endothelial cell functions

**Figure 1:**
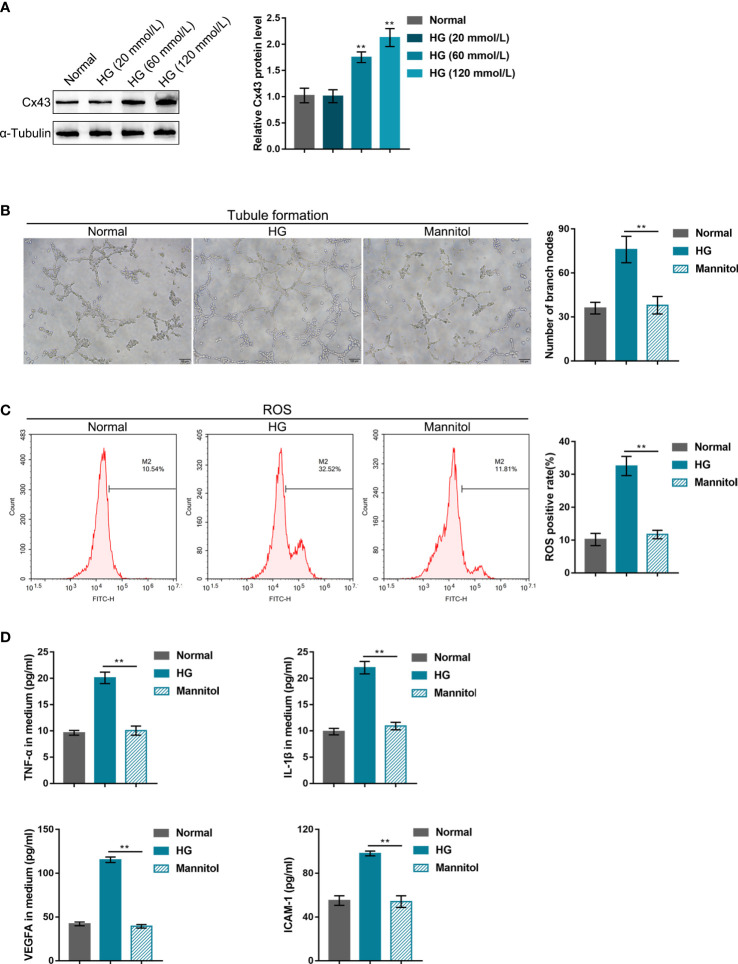
High-glucose (HG) impairs human retinal endothelial cell functions **(A)** hRECs were stimulated with gradient concentrations of glucose (20, 60, or 120 mmol/L) and examined for connexin 43 (Cx43) protein level using Immunoblotting. hRECs were subsequently stimulated with HG (60 mmol/L) or mannitol (60 mmol, to exclude the osmotic effect) and examined for tubule formation on Matrigel **(B)**; ROS release using flow cytometry **(C)**; the content of tumor necrosis factor alpha (TNF-α), interleukin-1β (IL-1β), vascular endothelial growth factor A (VEGFA), and intercellular adhesion molecule 1 (ICAM-1) in culture medium using ELISA kits **(D)**. n = 3, **p<0.01 compared with the normal group.

For the minimum HG dose selection, hRECs were stimulated with gradient concentrations of glucose (20, 60, or 120 mmol/L) and examined for Cx43 protein level. As indicated by the results, 60 or 120 mmol/L HG dramatically increased the Cx43 protein level relative to the normal culture medium (**Figure 1A**). Therefore, 60 mmol/L HG was selected for the subsequent experiments. In the meantime, a mannitol treatment group (60 mmol/L) was set to exclude the osmotic effect. As shown in **Figures 1B-D**, mannitol treatment caused no significant alterations in cell phenotypes, including tubule formation, ROS release, and the levels of TNF-α, IL-1β, VEGFA, and ICAM-1 in the culture medium compared with the control group. After 60 mmol/L HG stimulation, hREC functions were examined. It was observed that HG stimulation significantly promoted hREC tubule formation (**Figure 1B**), ROS release (**Figure 1C**), and TNF-α, IL-1β, VEGFA, and ICAM-1 levels in the culture medium (**Figure 1D**).

### Effects of Cx43 knockdown or overexpression on HG-induced angiogenesis

**Figure 2:**
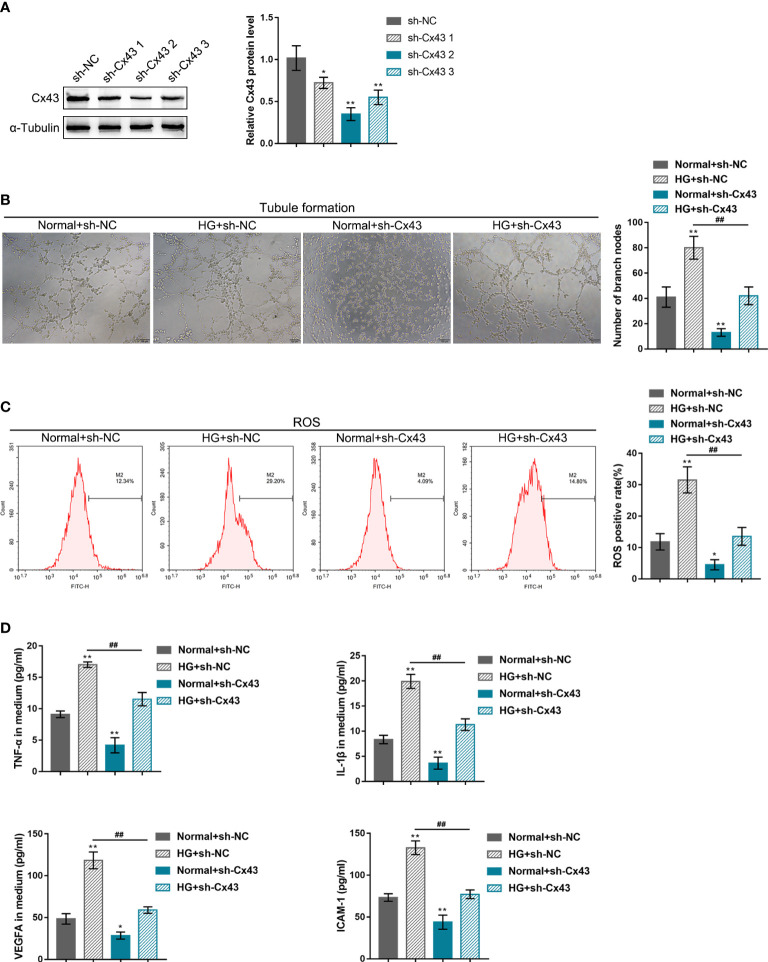
Effects of *Cx43* knockdown on HG-induced angiogenesis **(A)** Cx43 knockdown in hRECs was achieved through the transfection of the vector containing small hairpin RNA targeting *Cx43* (sh-Cx43#1/2/3); *Cx43* knockdown was confirmed using Immunoblotting. hRECs were subsequently transfected with sh-Cx43#2 (with better knocking down efficiency), cultured in normal or 60 mmol/L HG medium, and examined for tubule formation on Matrigel **(B)**; ROS release using flow cytometry **(C)**; the content of TNF-α, IL-1β, VEGFA, and ICAM-1 in culture medium using ELISA kits **(D)**. n = 3, *p < 0.05, **p < 0.01 compared with normal+sh-NC group; ^##^p < 0.01 compared with HG+sh-NC group.

**Figure 3:**
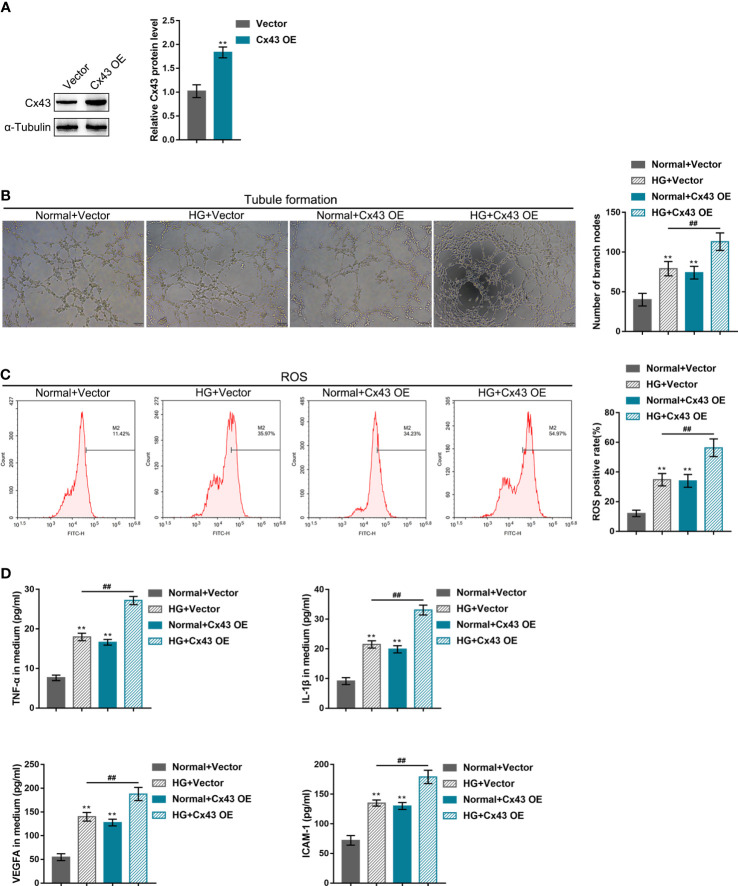
Effects of *Cx43* overexpression on HG-induced angiogenesis **(A)** *Cx43* overexpression in hRECs was achieved through transfection of the vector containing *Cx43* gene coding sequence (Cx43 OE); *Cx43* overexpression was confirmed using Immunoblotting. hRECs were subsequently transfected with Cx43 OE, cultured in normal or 60 mmol/L HG medium, and examined for tubule formation on Matrigel **(B)**; ROS release using flow cytometry **(C)**; the content of TNF-α, IL-1β, VEGFA, and ICAM-1 in culture medium using ELISA kits **(D)**. n = 3, **p < 0.01 compared with normal+vector group; ^##^p < 0.01 compared with HG+vector group.

hRECs were transfected with the vector containing shRNA targeting *Cx43* (sh-Cx43#1/2/3) or *Cx43* coding sequence (Cx43 OE) for *Cx43* knockdown/overexpression to investigate *Cx43*-specific effects on HG-induced angiogenesis. *Cx43* knockdown or overexpression was confirmed using Immunoblotting (**Figures 2A**, **3A**), and sh-Cx43#2 was selected for the subsequent experiments. The transfected cells were cultured in the normal or HG medium and examined for angiogenesis. The results revealed that HG stimulation promoted hREC tubule formation (**Figures 2B**, **3B**), elevated hREC ROS release (**Figures 2C**, **3C**), and increased TNF-α, IL-1β, VEGFA, and ICAM-1 levels in the culture medium (**Figures 2D**, **3D**). Under normal culture conditions, *Cx43* knockdown remarkably inhibited hREC tubule formation (**Figure 2B**), reduced hREC ROS production (**Figure 2C**), and decreased TNF-α, IL-1β, VEGFA, and ICAM-1 levels in the culture medium (**Figure 2D**); *Cx43* overexpression caused the opposite effects to *Cx43* knockdown on hREC angiogenesis, as manifested by the promoted tubule formation (**Figure 3B**), elevated ROS release (**Figure 3C**), and increased TNF-α, IL-1β, VEGFA, and ICAM-1 levels in the culture medium (**Figure 3D**). The promoting effects of HG on hREC angiogenesis were partially eliminated after *Cx43* knockdown (**Figures 2B–D**), whereas even magnified after *Cx43* overexpression (**Figures 3B–D**). From all above, *Cx43* knockdown attenuates, whereas *Cx43* overexpression promotes HG-induced hREC angiogenesis. Furthermore, the effects of oxidative stress alone or combined with an antioxidant (N-Acetyl Cysteine, NAC) on hRECs were determined to investigate whether Cx43 could be regulated by ROS. **Figure S2** shows that ROS release was dramatically elevated by HG stimulation and partially decreased by NAC treatment (**Figure S2A**); in the meantime, HG-induced Cx43 protein levels elevation was partially reduced by NAC treatment (**Figure S2B**). Therefore, Cx43 might be regulated by ROS.

### *In-vivo* effects of Cx43 knockdown on DR mouse model

**Figure 4:**
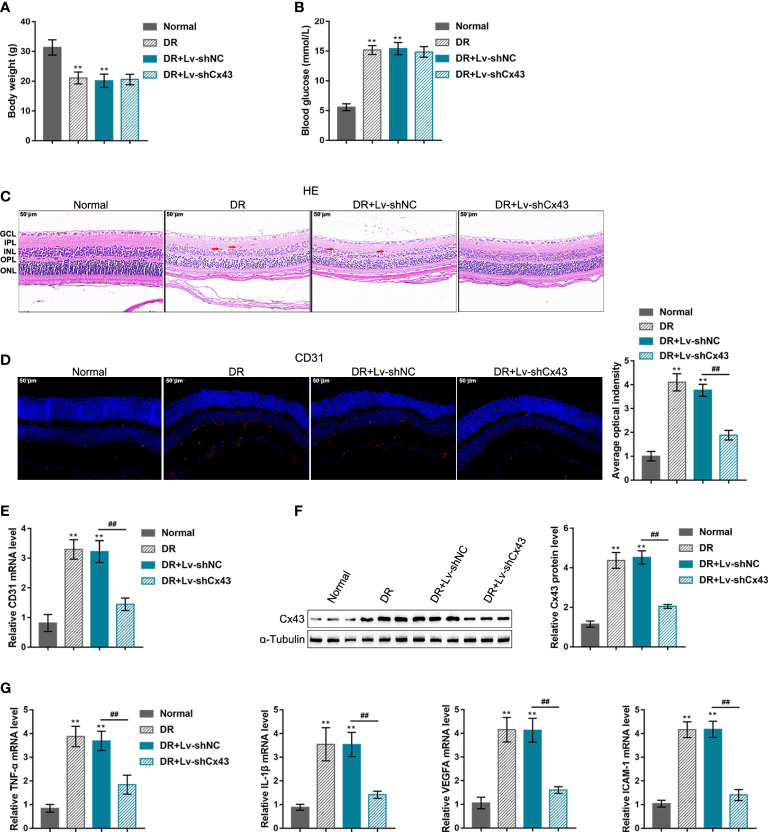
*in-vivo* effects of *Cx43* knockdown on the DR mouse model DR model was induced in C57BL/6 J mice and lentivirus infection as previously described. **(A, B)** Body weight and blood glucose of mice in different groups were determined. **(C)** The histopathological alterations in the retina were examined using H&E staining. ganglion cell layer (GCL); inner plexiform layer (IPL); inner nuclear layer (INL), outer plexiform layer (OPL); outer nuclear layer (ONL). Red arrows indicated microvessels. **(D)** CD31 levels in the retina were examined using IF staining. **(E)** The mRNA expression of CD31 in the retina was determined using qRT-PCR. **(F)** The protein levels of Cx43 in the retina were determined using Immunoblotting. **(G)** The mRNA expressions of TNF-α, IL-1β, VEGFA, and ICAM-1 in tissue samples were examined using qRT-PCR. n = 6, **p < 0.01 compared with normal group; ^##^p < 0.01 compared with DR+Lv-shNC group.

The DR mouse model was induced through sh-Cx43 lentivirus infection into C57BL/6 J mice as previously described to investigate the *in-vivo* effects of Cx43 on retinal neovascularization during DR (29). The body weight and blood glucose of mice in each group were monitored; **Figures 4A**, **B** shows that mice in the DR and DR + Lv-sh-NC groups saw significantly decreased body weight and elevated blood glucose, whereas intravitreally injection of Lv-sh-Cx43-induced *Cx43* knockdown in retina did not affect mice body weight and reduced blood glucose. As indicated by H&E staining results, the disorder in cell layers reduced thickness of the retina and microvessels in the inner plexiform layer (IPL) were observed in the DR and DR + Lv-sh-NC groups, which were partially improved after Lv-sh-Cx43 infection (**Figure 4C**). CD31 levels in the retina (a marker of retinal neovascularization) were examined using IF staining, and its mRNA expression was determined using qRT-PCR. The results revealed that CD31 level was markedly elevated in the DR and DR + Lv-sh-NC groups whereas partially reduced after Lv-sh-Cx43 infection (**Figures 4D**, **E**). Cx43 knockdown in the retina was also confirmed using Immunoblotting; **Figure 4F** shows that the levels of Cx43 were significantly elevated in the DR groups, whereas effectively decreased by Lv-sh-Cx43 infection (**Figure 4F**). *TNF-α*, *IL-1β*, *VEGFA*, and *ICAM-1* mRNA levels in tissues were detected, which were observed to be remarkably upregulated in the DR and DR + Lv-sh-NC groups whereas partially downregulated after Lv-sh-Cx43 infection (**Figure 4G**).

## Discussion

In this study, high glucose increased *Cx43* expression in hRECs in a dose-dependent manner. Compared with hRECs under normal culture conditions, HG-stimulated hRECs showed notably promoted tubule formation, ROS release, and TNF-α, IL-1β, VEGFA, and ICAM-1 levels in the culture medium. These HG-induced alterations were further magnified after *Cx43* overexpression, whereas partially eliminated after *Cx43* knockdown. Finally, in the DR mouse model, impaired retina structure, increased CD31 level (a marker of retinal neovascularization), and upregulated mRNA levels of TNF-α, IL-1β, VEGFA, and ICAM-1 were observed; *in-vivo Cx43* knockdown partially reversed these phenomena s.

During DR, pathological angiogenesis is one of the leading causes of permanent blindness in people of all ages. One of the key pathological conditions contributing to these vision-threatening retinopathies is retinal neovascularization (30, 31). The term angiogenesis was initially utilized to explain the sprouting of new capillaries from the pre-existing postcapillary venules (32). As aforementioned, angiogenesis is a complex process, in which tubule formation by endothelial cells is a critical event (32, 33). In the present study, HG stimulation increased Cx43 levels in hRECs in a dose-dependent manner, which suggested the potential role of Cx43 in hREC angiogenesis. As expected, HG stimulation dramatically promoted hREC tubule formation, further enhanced after *Cx43* overexpression, whereas partially attenuated after *Cx43* knockdown. Moreover, as evidenced previously, *Cx43* siRNA-treated EPCs lose their therapeutic angiogenic potential in a mouse hindlimb ischemia model (25). Consistently, the findings in this study further evidenced the promotive role of Cx43 in hREC angiogenesis.

It’s well-established that excessive ROS produced under various physiological and pathological conditions [such as inflammation (34) and ischemia (35)] may lead to the metabolic imbalance of oxidative stress-generated reactive intermediates and multiple disorders, including DR (36, 37). A previous study has pointed out that hypoxia-inducible factor (HIF)-1α-mediated hypoxic response is tightly linked to hypoxia-induced ROS production (38). Accumulating studies have shown that VEGF and ROS play key roles in vascular pathophysiology (39, 40). VEGF plays an essential role in the disorder, and its interaction with neutralizing molecules represents a significant advancement in diabetes treatment (41). VEGF could promote the proliferation and migration of vascular endothelial cells, and elicit blood vessel permeability. Hypoxia-induced increase of VEGF has emerged as a major driver of neovascularization in DR (42). In this study, HG-treated hRECs presented elevated ROS release and increased VEGFA levels in the culture medium. Similarly, HG-induced increase of ROS and VEGFA levels were both reduced after *Cx43* knockdown, which suggested that *Cx43* knockdown inhibited HG-induced angiogenesis in hRECs.

As a typical characteristic of diabetes, chronic inflammation also occurs in the eyes (43). Elevated levels of IL-1β and TNF-α are tightly implicated in DR (44). Reportedly, pro-inflammatory cytokines result in the breakdown of the blood-retinal barrier in DR through various molecular mechanisms in the retinal vasculature (45, 46). As a result, the reduced expressions of pro-inflammatory cytokines may show vascular advantages in DR. A previous study has pointed out that Cx43 promotes inflammasome activation and the development of renal inflammatory cell damage through intracellular redox regulation (47). Cx43 has been reported to be involved in both cytokine and immunoglobulin secretion in diseased human corneas; Cx43 in macrophages of distinct tissues could partially enhance the secretion of pro-inflammatory cytokines (48). In this study, HG stimulation dramatically elevated IL-1β and TNF-α levels, which were further amplified after *Cx43* overexpression whereas partially reduced after *Cx43* knockdown. The above data indicated that Cx43 knockdown could improve HG-induced retinal inflammation.

Furthermore, the above findings were further evidenced in an STZ-induced DR mouse model. In the DR mouse model, impaired retina structure, increased CD31 level (a marker of retinal neovascularization), and upregulated mRNA expression levels of TNF-α, IL-1β, VEGFA, and the ICAM-1 were partially abolished after *in-vivo* Cx43 knockdown. In summary, Cx43 knockdown could inhibit hREC angiogenesis and inflammation, therefore improving DR symptoms in the DR mouse model.

Concerning the possible mechanism of Cx43 function on angiogenesis and inflammation, endothelial cells in the retina are strongly stimulated by the coexistence of hyperglycemia and hypoxia, increasing *VEGF* expression (49). Cx43 has been reported to regulate the production of HIF-1 to control VEGF-induced angiogenesis (50). The HIF-1α response to hypoxia is associated with hypoxia-induced production of ROS (38), and ROS triggers the induction of VEGF (51–53). Since supplementary experiments indicate that the elevation of Cx43 protein levels was associated with the ROS accumulation, the mechanism of Cx43 regulating pro-inflammatory factors and angiogenesis might be related to the coexistence of hyperglycemia and hypoxia and oxidative stress, which needs further investigations in our future studies.

## Data availability statement

The raw data supporting the conclusions of this article will be made available by the authors, without undue reservation.

## Ethics statement

The animal study was reviewed and approved by ethic committee of Xiangya Hospital of Central South University.

## Author contributions

WS: performed the experiments, wrote manuscript. ZM: Performed the experiments. JL: Supervision, edited manuscript. All authors contributed to the article and approved the submitted version.

## Funding

This study was supported by Natural Science Foundation of Hunan Province, China (2021JJ40862) and Natural Science Foundation of Changsha City, China (kq2014249).

## Conflict of interest

The authors declare that the research was conducted in the absence of any commercial or financial relationships that could be construed as a potential conflict of interest.

## Publisher’s note

All claims expressed in this article are solely those of the authors and do not necessarily represent those of their affiliated organizations, or those of the publisher, the editors and the reviewers. Any product that may be evaluated in this article, or claim that may be made by its manufacturer, is not guaranteed or endorsed by the publisher.

## Supplementary material

Supplementary Figure 1 Images with the fluorescein signal combined with the fsc/ssc scatter.

Supplementary Figure 2 Effects of 60 mmol/L HG and antioxidant (N-Acetyl Cysteine, NAC, 1mmol/L) on ROS release **(A)** and Cx43 expression **(B)** in hRECs.
