# Supplementary figures and images for: Connexin 43 (Cx43) regulates high-glucose-induced retinal endothelial cell angiogenesis and retinal neovascularization
Source: Front Endocrinol (Lausanne). 2022 Sep 2;13:909207. doi: 10.3389/fendo.2022.909207 (PMC9478119; doi:10.3389/fendo.2022.909207)

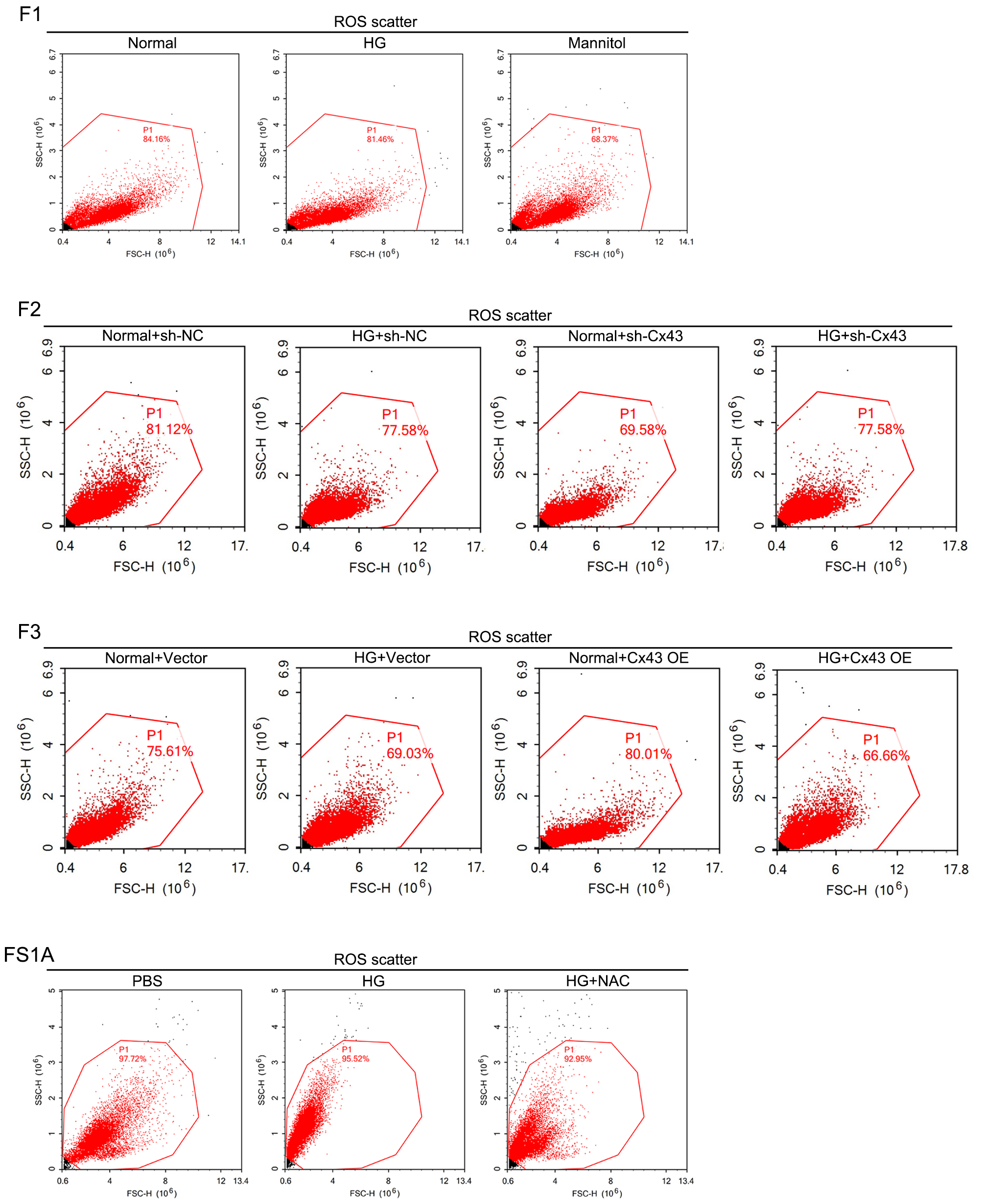

Supplement: Supplementary Figure 1 — Images with the fluorescein signal combined with the fsc/ssc scatter. [file Image_1.tif]

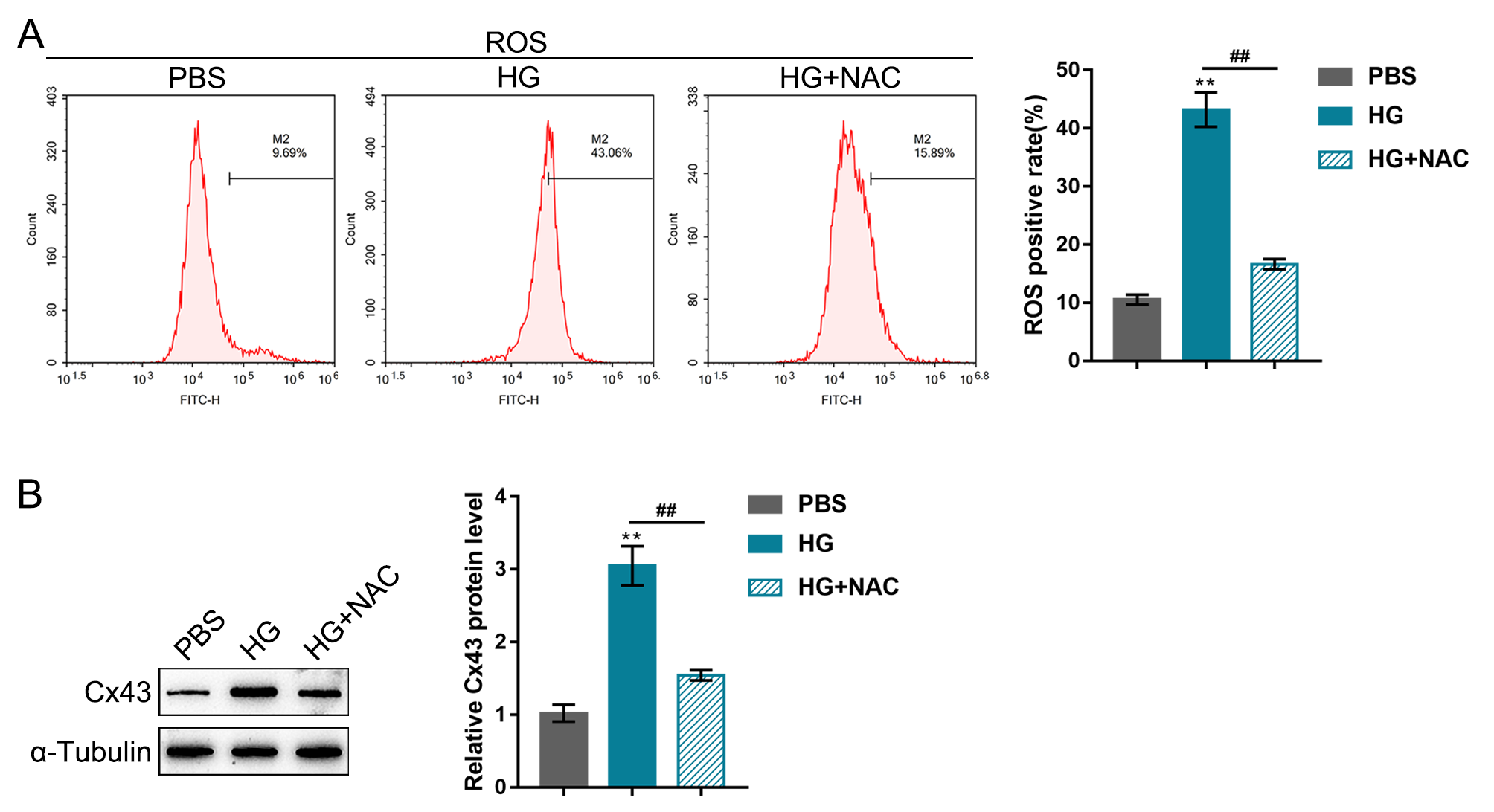

Supplement: Supplementary Figure 2 — Effects of 60 mmol/L HG and antioxidant (N-Acetyl Cysteine, NAC, 1mmol/L) on ROS release (A) and Cx43 expression (B) in hRECs. [file Image_2.tif]
